# Quantitative Multiparameter Evaluation of Vacuoles in Intraocular Lenses Employing a High-Magnification Digital Microscopy Method

**DOI:** 10.1155/2019/7929014

**Published:** 2019-08-04

**Authors:** Vincent Spiezio, Bennett N. Walker, Don Calogero, Ilko K. Ilev

**Affiliations:** ^1^Optical Therapeutics and Medical Nanophotonics Laboratory, Office of Science and Engineering Laboratories, Center for Devices and Radiological Health, U.S. Food and Drug Administration, Silver Spring, Maryland 20993, USA; ^2^Office of Device Evaluation, Center for Devices and Radiological Health, U.S. Food and Drug Administration, Silver Spring, Maryland 20993, USA

## Abstract

As small imperfections with micrometric sizes, fluid-filled vacuoles, also referred to as glistenings, in intraocular lenses (IOLs) have been known to induce significant unwanted light scattering that in several cases presumably cause complaints and sometimes lead to IOL explantation and replacement. This unwanted scatter is of particular concern for patients viewing bright light in reduced-light conditions such as when driving at night, as the scattered light toward the retina can cause temporary blindness. In this study, we have developed and implemented an accurate test methodology based on a high-magnification digital microscopy approach for quantitative multiparameter evaluation and classification of IOL vacuoles depending on their critical optical characteristics including vacuole size, density, shape, and orientation within the IOL material. Using the multiparameter database developed by evaluating vacuole characteristics, we established a classification grading system that can be used to evaluate vacuole effects on light scattering.

## 1. Introduction

Intraocular lens (IOL) implantation to treat cataracts and aphakia is one of the most commonly performed surgical procedures, with about 3.6 million per year in the United States alone [1, 2]. Since IOLs were first introduced, new IOL designs and materials have been developed and utilized to improve their optical properties and clinical outcomes. However, some newer designs and materials used in IOLs have an increased tendency to form small, micrometer-sized, fluid-filled vacuoles within the bulk of the IOL [3–18]. These vacuoles, also commonly referred to as glistenings, have been shown to reduce contrast sensitivity (CS) and visual acuity (VA) [13, 14, 17, 19–21], in some cases requiring IOL explantation [17, 21].

However, in many cases, vacuoles have been found to have a negligible impact on visual function [5, 7, 10]. A recent study by van den Berg [22] demonstrated a lack of correlation between straylight and VA, indicating rather that small aberrations within the lens are more responsible for loss of VA. Wider angle straylight, on the other hand, most commonly causes a loss of CS, which is not as noticeable or as great a cause for concern. In some cases, however, straylight can also cause disability glare, so further distinctions need to be made between the angular degree of scattered light and clinical effects.

Currently, one of the most commonly employed methods to classify vacuole severity is the use of a grading scale, in which IOLs are classified based on the number of vacuoles counted or observed [6, 7, 13, 14, 23, 24]. This is usually done through slit-lamp imaging, but in one case, Scheimpflug imaging was used [23]. The grading scales generally range from grade 0, having no or trace vacuoles, to the highest grade, usually either 3 or 4, having extremely severe vacuoles. Various test methods have been employed to measure the clinical and nonclinical effects of vacuoles and light scatter in general, including subjective patient reports and VA tests [7, 10, 11, 13, 14, 16, 17, 19–21], integrating spheres and transmission measurements [16, 21, 24–26], C-Quant straylight meter measurements [18, 20, 27–29], and *in vivo* slit-lamp and Scheimpflug tests and measurements [7, 10, 11, 13–17, 19–21, 23]. In addition, two different methods have been independently developed to physically scan around the back of the IOL to measure forward scattered light [26, 27, 30, 31]. However, these methods and reported studies have only been correlated with vacuoles solely in terms of vacuole size and density.

While vacuoles generally manifest as rounded structures, they also may appear oblong- or rod-like when observed under a microscope. This geometric feature has been studied in a previously published work, illustrating that it results from either flattened ellipsoid or disc-shaped vacuoles oriented at a non-normal angle to the IOL plane [9]. However, it is conventionally assumed in most previously published studies on characterizing individual vacuoles that all vacuoles are round and behave as spheres while not considering the general shape and orientation aspect that causes a significant portion of vacuoles appear either oblong- or rod-like [4, 8, 20, 32]. None of the current grading systems account for specific vacuole shape and orientation, rather looking only at the density and effective diameter. This minimally classified vacuole characteristic may have an additional effect on light-scattering properties of IOLs and merit further investigation.

In the present study, we developed and implemented an accurate test methodology based on a high-magnification digital microscopy approach to quantitatively evaluate and classify IOL vacuoles depending on their critical multiparameter optical characteristics including vacuole size, density, shape, and orientation. This quantitative multiparameter evaluation method was used to establish a new classification grading system that can potentially be employed to evaluate vacuole effects on light scattering.

## 2. Materials and Methods

**Figure 1:**
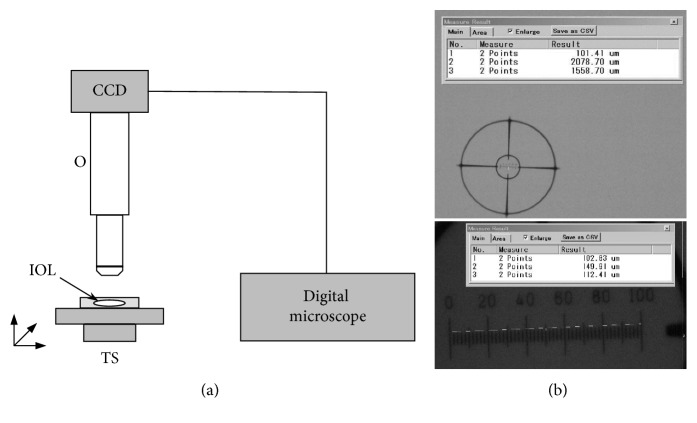
(a) Schematic diagram of the high-magnification digital microscopy-based approach for quantitative characterization of IOL vacuoles: O, high-magnification (75x to 5000x) microscope objective; CCD, digital camera; IOL, test IOL sample placed at *in situ* simulation conditions; TS, *x*-*y*-*z* translational stage. (b) 100 *μ*m calibration scale with a minimum spatial resolution of 2 *μ*m measured with the digital microscope at 150x (upper picture) and 2000x (lower picture) magnification, along with the measured FOV.

To quantitatively evaluate multiparameter optical characteristics of light-scattering vacuoles in IOLs (such as vacuole size, density, shape, and orientation), we developed an experimental test methodology using a high-magnification digital microscopy approach illustrated in Figure 1(a). The test system includes a digital optical microscope (VHX-100, Keyence, Inc.) that provides some advanced features essential for this study such as high magnification in the range of 75x to 5000x, a large objective working distance of 3 mm to 48 mm, and a submicron spatial resolution for precisely measuring IOL vacuole size, density, shape, and orientation.

Before investigation of IOL vacuoles, the accuracy of the microscope measurements was validated. To do this, a 100 *μ*m calibrated microscopic scale with a minimum spatial resolution of 2 *μ*m was measured with a digital microscope at 150x and 2000x magnification, along with the measured field of view (FOV), as illustrated in Figure 1(b). Experimentally assessing the measurement tool on the digital microscope provides an accuracy within 2% and 3% for 150x and 2000x magnification, respectively.

**Figure 2:**
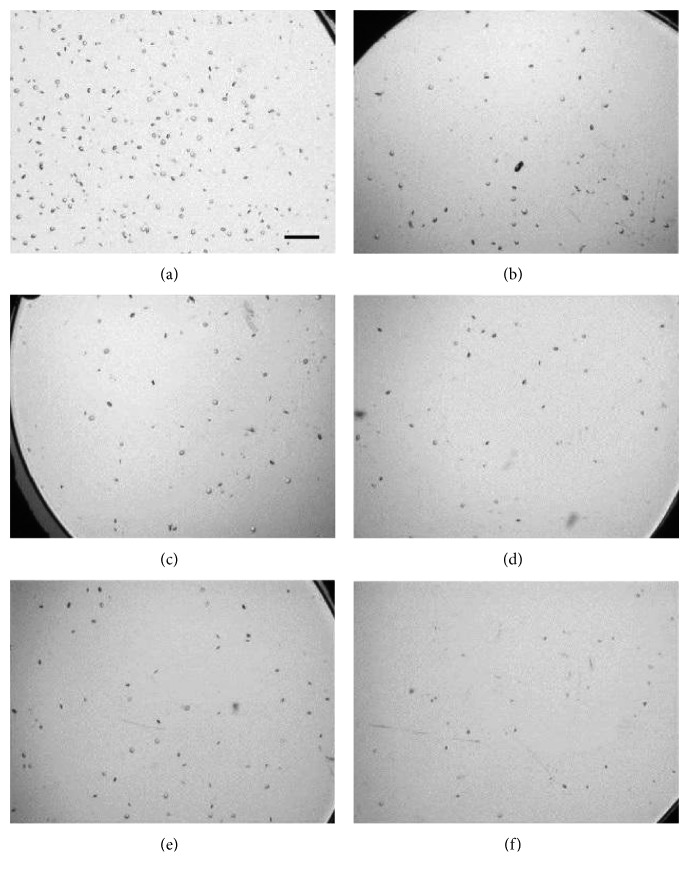
Images of IOL #1 (a), #2 (b), #3 (c), #4 (d), #5 (e), and #6 (f) at 75x magnification. Scale bar, 500 *μ*m.

**Figure 3:**
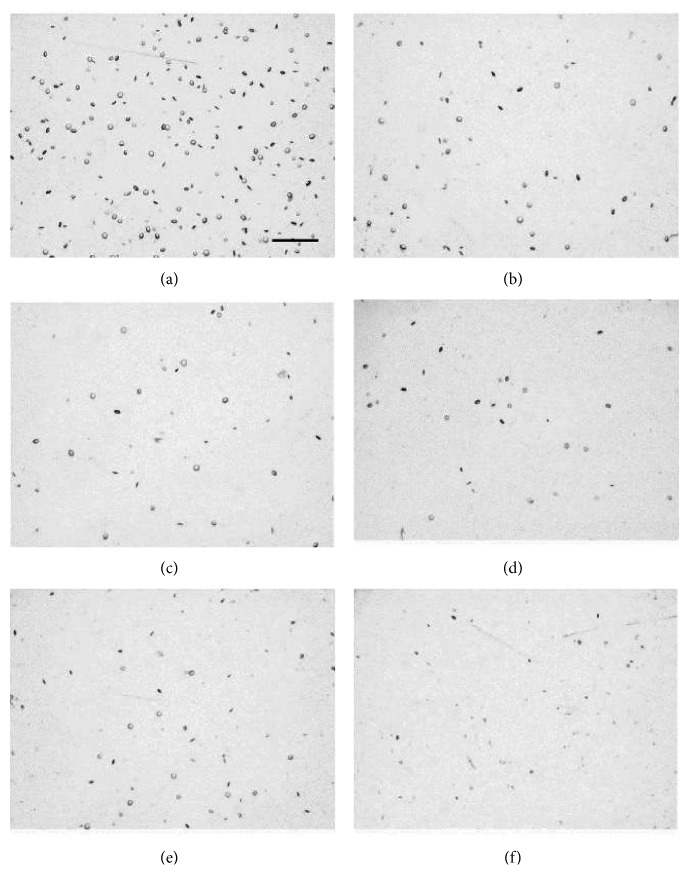
Images of IOL #1 (a), #2 (b), #3 (c), #4 (d), #5 (e), and #6 (f) at 100x magnification. Scale bar, 500 *μ*m.

**Figure 4:**
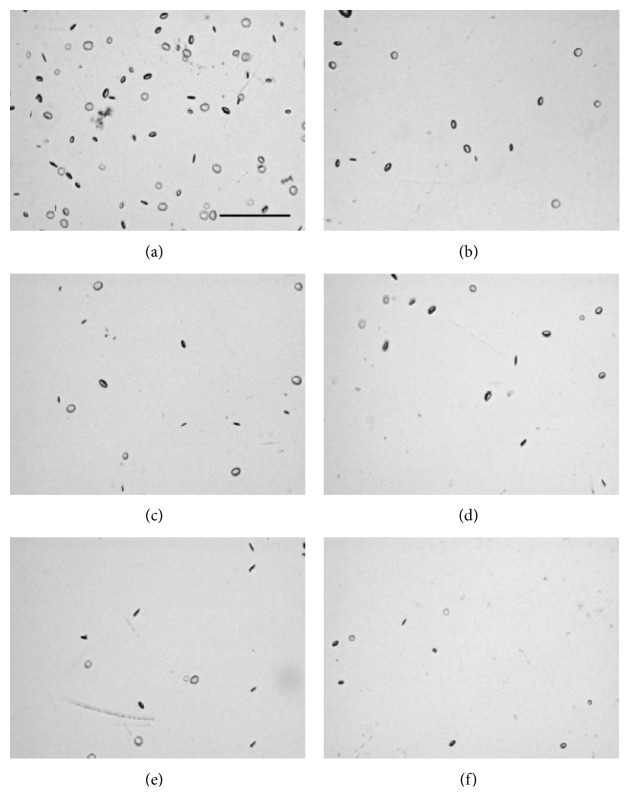
Composite images of IOL #1 (a), #2 (b), #3 (c), #4 (d), #5 (e), and #6 (f) at 150x magnification. Scale bar, 500 *μ*m.

For vacuole characterization, seven monofocal IOL test samples were acquired from the manufacturer with varying degrees of induced vacuoles that had been naturally generated while stored in a balanced salt solution. The material and IOL models were not specified, but these variables have no effect on the methodology or conclusions of this study [24]. Of these lenses, IOLs #1–6 contain fewer than 500 vacuoles, with vacuole diameters generally between 30 and 50 *μ*m, which is atypical of IOL vacuoles. The seventh IOL contains approximately 300,000 vacuoles roughly 5 *μ*m across, which is more typical, and is used to validate the methodology used to characterize vacuoles in IOLs #1–6. IOLs were placed in a saline-filled cuvette simulating *in situ* conditions and then positioned under the digital microscope objective on a precise *x*-*y*-*z* translational stage. The IOL vacuoles in IOLs #1–6 were imaged and evaluated using the digital microscope with a magnification of 75x (Figures 2(a)to 2(f) for IOL test samples #1 to #6, respectively), 100x (Figures 3(a)to 3(f) for IOL test samples #1 to #6, respectively), and 150x (Figures 4(a)to 4(f) for IOL test samples #1 to #6, respectively).

For IOLs #1–6, vacuole evaluation was performed using two different methods. For the first IOL evaluation method, referred to as “Method 1,” multiple images were taken at different locations on the IOL at 100x and 150x magnification and later analyzed to determine the vacuole density as well as various geometrical characteristics. For images taken at 150x magnification, because of the reduced depth of field (DOF) relative to 75x and 100x, multiple images had to be taken at different depths throughout the IOL and were combined to create a composite image. Using a precise measurement tool on the digital microscope, each individual vacuole in the 150x composite images was counted, and each vacuole's major and minor axes were measured.

For the images taken at 100x magnification, the vacuoles were only counted, without measuring them, as the vacuoles appear too small to reliably measure but are large enough to identify. In addition, at 100x magnification, the measured FOV is approximately 3.64 mm × 2.65 mm, giving a total area of about 9.4 mm^2^. This corresponds to about 1/3 of the total surface area of a 6 mm diameter IOL. At 150x magnification, the FOV is approximately 2.08 mm × 1.56 mm or about 3.2 mm^2^, which corresponds to only about 1/9 of the total IOL surface area. This smaller FOV requires taking more images throughout the IOL to accurately infer the density of vacuoles. Therefore, 100x magnification is used predominantly for more precisely determining the overall density measurements for each IOL.

**Figure 5:**
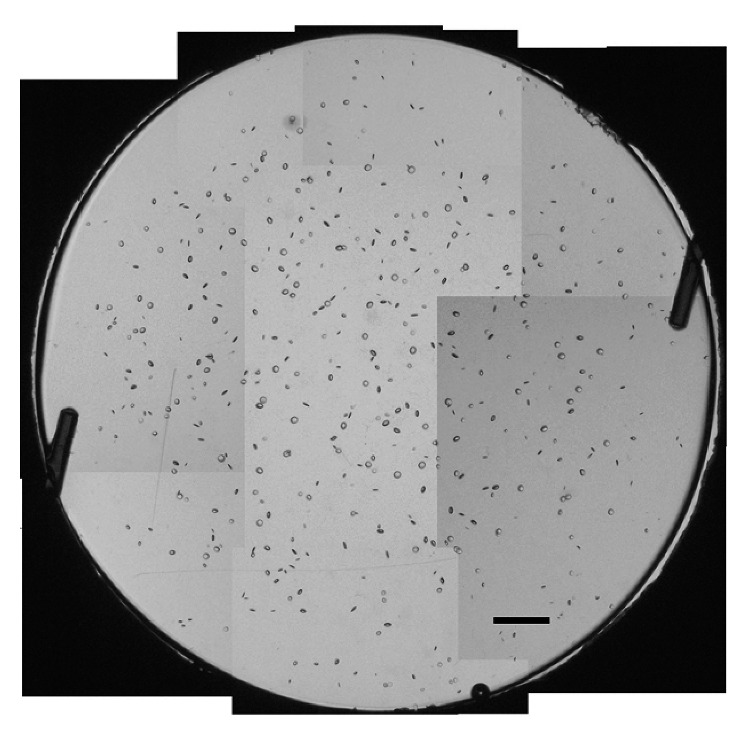
Stitched composite image of IOL #1 at 100x magnification. Scale bar, 500 *μ*m.

For the second IOL evaluation method, referred to as “Method 2,” digital microscope imaging to identify and measure each individual vacuole in each IOL was performed. Because the lowest available magnification of 75x used in the study is not able to image the entire IOL at once, composite images of each IOL were created. Images were taken throughout IOLs #1–6 using 100x magnification, so that a composite image of the entire IOL could be stitched together. Each vacuole was then identified, and its dimensions were measured using ImageJ image processing software. Due to the differences in background brightness and shading throughout the images, as seen in Figure 5, a direct automatic measurement using ImageJ was unfeasible and unreliable, so each vacuole was identified and measured manually. A typical stitched composite image of IOL #1 is shown in Figure 5.

**Figure 6:**
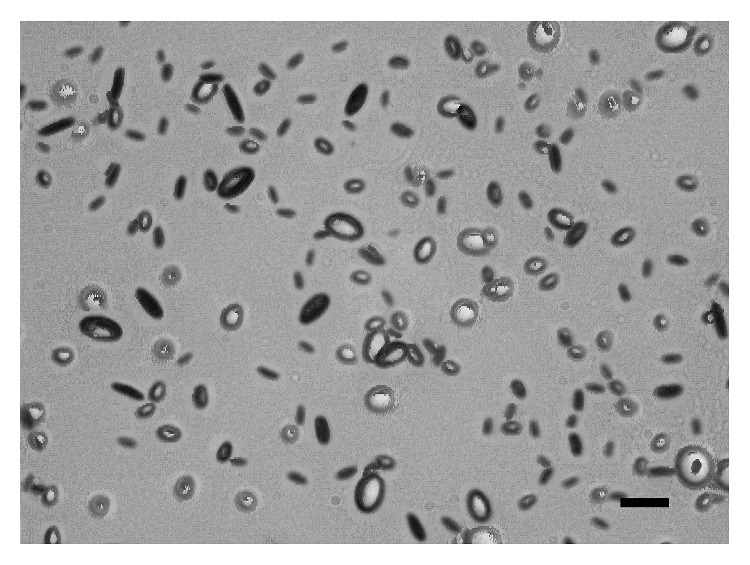
2000x magnification composite image of IOL #7. Scale bar, 10 *μ*m.

For IOL #7, imaging and analysis was done similarly to IOLs #1–6, but with some key differences. Because vacuoles were much smaller and denser, counting and measuring each individual vacuole as was done in Method 2 is impractical, so a modified version of Method 1 was used exclusively. Three composite images were taken near the center of IOL #7 at 2000x magnification due to the low DOF, and each vacuole was manually measured and characterized using a measurement tool on the digital microscope. One of these images is shown in Figure 6.

As performed for IOLs #1–6, each vacuole in the three images taken of IOL #7 was counted and measured to demonstrate the validity of this method for smaller size vacuoles. The total number of vacuoles present, as well as overall vacuole characteristics, was inferred based on analysis of the three images, assuming consistent distribution throughout the lens.

**Figure 7:**
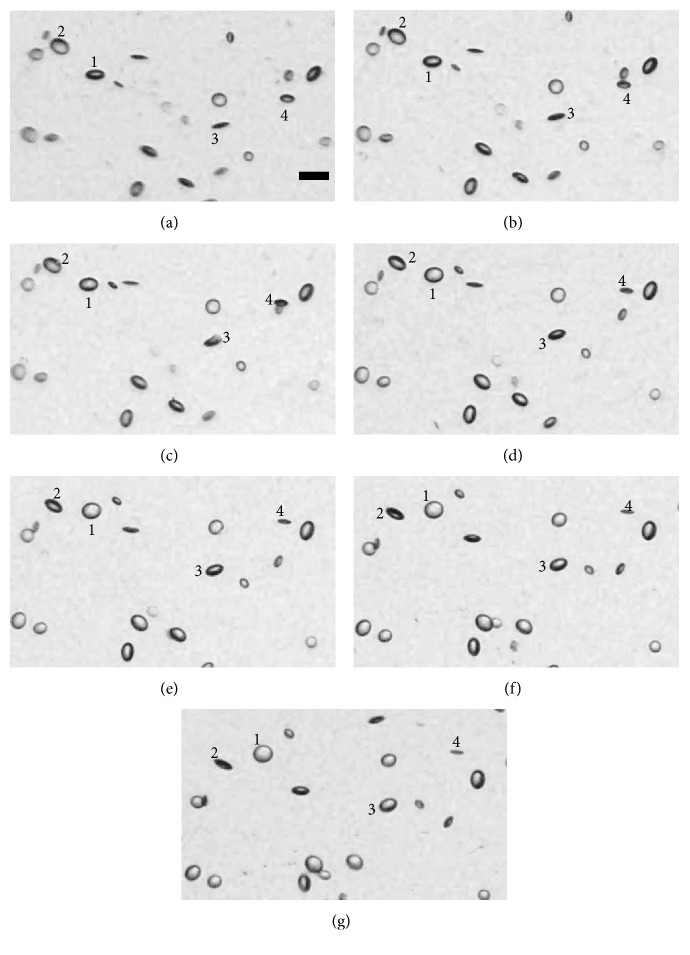
Vacuoles present in IOL #1 taken with the IOL angled −40° (a), −30° (b), −20° (c), −10° (d), 0° (e), 10° (f), and 20° (g) from a normal incidence angle of observance, imaged at 100x magnification. Scale bar, 100 *μ*m.

One previous study demonstrated that vacuoles are in fact either discs or flattened ellipsoids [9]. However, as most published studies gloss over this important vacuole characteristic, we have performed an independent investigation on it. For IOL #1, 7 images are taken at 100x magnification, tilting the IOL from −40° to 20° from normal incidence in 10° angular increments. To examine the effects that the angle of observance has on the apparent shapes of vacuoles, a small section of each image is looked at. Figures 7(a)–7(g) show images of vacuoles present in IOL #1 taken at −40°–+20°, respectively, in 10° increments.

As shown in Figure 7, four different vacuoles are numbered to assist in determining the changes in apparent shape that occur when changing the angle of observation. At −40° (Figure 7(a)), vacuoles labeled 1 and 3 appear elongated. As the angle of observation increases to 20° (Figure 7(g)), these vacuoles appear to widen, becoming more rounded. Conversely, the vacuoles labeled 2 and 4 are rounded at −40°, and by 20°, they are further elongated. This helps to confirm that vacuoles manifest as either discs or flattened ellipsoids and that vacuoles that appear elongated are simply being viewed at a steep angle.

Knowing that vacuoles are in fact either discs or flattened ellipsoids, despite their apparent oblong- or rod-like appearance, a method was developed to classify vacuoles based on their rotation angle with respect to the IOL. For each vacuole, a rotation angle compared to the normal incidence angle was estimated, with 0° being in the same plane as the IOL, appearing to be a perfect circle. The rotation angle was estimated using the following equation:

(1) $$\begin{array}{c} R=\;\;\cos^{-1}\frac{Y}{X}, \end{array}$$

where *X* is the measured major axis length, *Y* is the measured minor axis length, and *R* is the rotation angle. This dependence provides the approximate rotation of each vacuole from normal, knowing what the resulting apparent dimensions of the vacuoles appear to be. This calculated angle assumes that these vacuoles have a negligible thickness, so that when viewed at a steep angle, the primary surface being seen is the vacuole face and not the edge. In addition, it assumes that the vacuole is circular and not elliptical.

Five vacuole orientation groups are defined based on the approximate rotation angle of the individual vacuole. The rotation ranges are chosen based on the microscopic visual appearances of individual vacuoles, with each grouping assigned based on the calculated rotation. These five vacuole groups, defined based on the estimated rotation angle, are shown in Table 1.

**Figure 8:**
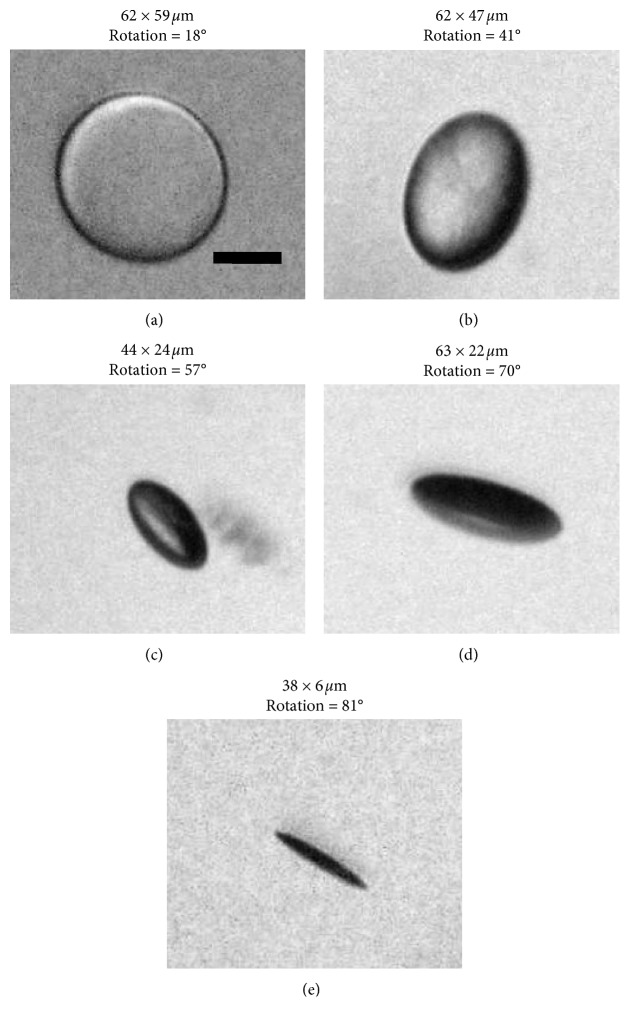
Representative examples of vacuoles in group 1 (a), group 2 (b), group 3 (c), group 4 (d), and group 5 (e) with their measured dimensions and calculated rotations, imaged at 450x magnification. Scale bar, 25 *μ*m.

To supplement the data gathered at 100x and 150x magnification, several individual vacuoles are imaged and measured at 450x magnification. The acquired data include images of vacuoles from each orientation group described in Table 1, and representative examples from each group are shown in Figure 8 (Figures 8(a)to 8(e) for vacuole group 1 to group 5, respectively). The 450x magnification used here was too high to acquire representative samples of vacuoles present in each IOL, but it was sufficient and necessary to acquire high-resolution images of individual vacuoles.

**Figure 9:**
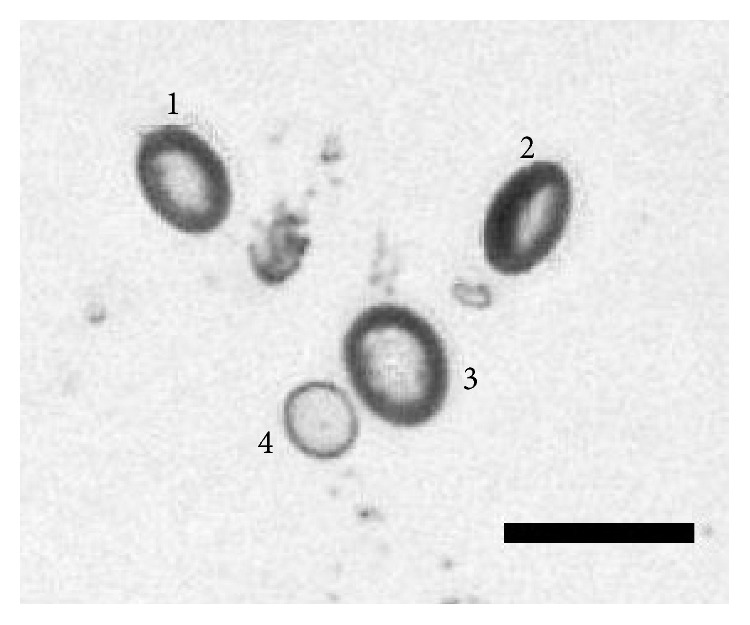
Four vacuoles from IOL #1 imaged at 150x magnification. Scale bar, 100 *μ*m.

To validate the accuracy and precision of the measurements taken, four individual vacuoles in IOL #1 were imaged four times at 150x magnification. These vacuoles were each measured, with the resulting tilt angle and groupings determined. These results were compared to each other to determine the repeatability of the methodology used here. These four representative vacuoles in IOL #1, numbered as 1–4, are shown in Figure 9.

## 3. Results

The total number of vacuoles in IOLs #1–6, as well as the average vacuole diameter, was measured and calculated for both IOL evaluation methods used. For vacuoles counted using Method 1, the overall number of vacuoles was estimated, assuming a uniform distribution of vacuoles throughout the IOL. This estimation also assumes some standard IOL geometric parameters including an IOL diameter of 6 mm, a center thickness of 0.7 mm, an edge thickness of 0.5 mm, and spherical front and back surfaces, giving an IOL volume of roughly 17 mm^3^. Estimation of total vacuole count also assumes a thickness of 0.7 mm for the 100x and 150x images in which vacuoles were counted. The vacuole counts for Method 2 represent the total number of vacuoles counted in the entire stitched composite image. These results are shown in Table 2.

Of the six IOL samples studied, IOL #1 had by far the highest number of vacuoles, with the remaining five IOLs having more similar numbers of vacuoles. This assessment was validated using the vacuole counts at both 100x and 150x magnification. Error was calculated based on the standard deviation of the vacuoles counted in multiple images of the same IOL. As expected, the standard deviation for the vacuoles counted at 100x magnification is significantly lower than that at 150x magnification, as a larger portion of the IOL is imaged at a time. In addition, the estimated vacuole count was more accurate at 100x magnification than at 150x magnification.

For each IOL imaged using Method 2, the diameter of each individual vacuole was measured, and the average vacuole diameter in each lens was calculated. In addition, for vacuoles imaged from individual 150x magnification images, as done in Method 1, the approximate average vacuole diameter including the standard deviation related to the estimated accuracy and precision of the diameter value was calculated. These are shown in Table 3.

**Figure 10:**
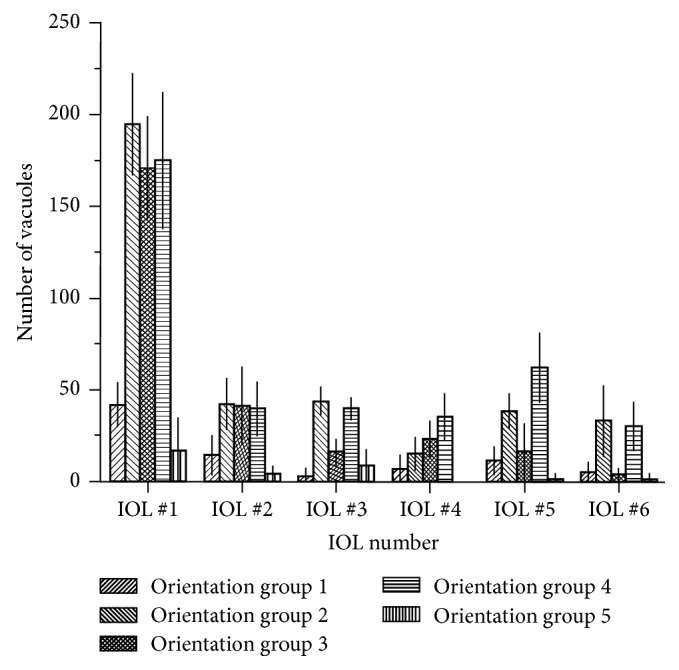
Estimated numbers of each vacuole orientation group in IOLs #1–6. Error bars represent standard deviations in estimated numbers.

**Figure 11:**
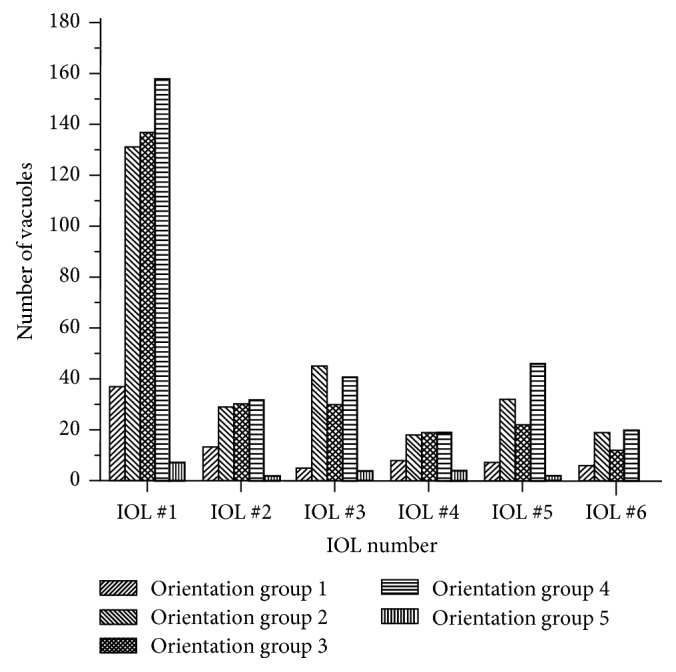
Counted number of each vacuole orientation group from stitched composite images of IOLs #1–6.

For each IOL, the overall number of vacuoles present from each orientation group was determined. For Method 1, the total number of vacuoles in each group was counted in each 150x image, to determine the average number of vacuoles in each group, as well as the standard deviations. This is shown in Figure 10. For Method 2, each individual vacuole in the stitched IOL image was identified, measured, and placed into one of the five groups. The number of vacuoles from each orientation group in each IOL using Method 2 is shown in Figure 11.

As expected, IOL #1 has more vacuoles from each group than the remaining IOLs. While IOLs #2–6 have similar overall densities of vacuoles, the distribution for the groups of vacuoles present vary greatly. As shown in Figures 10 and 11, the only consistent pattern seen is that vacuoles perfectly normal and perfectly perpendicular, represented, respectively, by groups 1 and 5, are relatively less common. For vacuole orientation groups 2, 3, and 4, there is no consistent pattern for which vacuoles are present more often than others.

Likewise, each vacuole present in the three images of IOL #7 was identified, measured, and characterized based on the groupings defined in Table 1. The total number of vacuoles counted throughout the three images and their proportion for each orientation group are shown in Table 4. As with IOLs #1–6, orientation groups 1 and 5 contain the least number of vacuoles, with most vacuoles being in groups 2–4.

Throughout the three images, an average of 195 vacuoles are counted and measured, with a standard deviation of 7 vacuoles between the three images taken. Each image taken at 2000x magnification has a total FOV of approximately 150 *μ*m × 110 *μ*m. Assuming a thickness of 0.7 mm, this corresponds to a volume of 1.2 × 10^−3^ mm^2^ or about 0.07% of the total volume of the IOL. Assuming a uniform thickness of 0.7 mm at the imaged location, the total number of vacuoles throughout the lens is estimated to be about 300,000.

As described previously, the repeatability of the study was also validated through replicate measurements of the four vacuoles shown in Figure 9. These vacuole measurements and the standard deviation between them are shown in Table 5. The propagation of this uncertainty to the calculated vacuole tilt angle is shown in Table 6.

As shown in Table 5, the standard deviation between measurements is quite small, never exceeding 5 *μ*m. However, because the vacuole sizes are also relatively small, this results in a percent standard deviation that generally falls between 5 and 10%. The propagation of this deviation to calculated orientation is shown in Table 6 and results in an uncertainty in orientation of nearly 10 degrees in one case.

## 4. Discussion

The six IOL samples tested have varying densities of vacuoles and varying distributions of the five orientation groups of vacuoles studied. To further evaluate the effects of vacuole orientation, we determined the theoretical distribution of vacuole orientations and compared these to the actual distributions. If a vacuole has an equal probability of assuming any orientation, the distribution should be directly based on the ranges used to define the five orientation groups defined in Table 1. Thus, the theoretical distribution that could be associated with each vacuole orientation group is illustrated in Table 7, alongside the experimental average distribution measured in IOLs #1–6.

As shown in Table 7, there is a discrepancy between the actual experimental and theoretical vacuole distributions, particularly in groups containing the smallest and largest rotation angles. This could be related to either a tendency of vacuoles to assume certain orientations within the lens, possibly due to polymer orientation, or from random and systematic error present in the evaluation methods used. As stated earlier, there is little known about vacuole orientation within IOLs, so further research needs to be done regarding whether the vacuole formation could be associated with a specific trend to orient in certain ways.

Another vacuole characteristic that may affect the calculated vacuole rotations is vacuole thickness. While due to some methodology and equipment limitations, objective classification of each vacuole's thickness was not experimentally performed, some specific assessment can be made regarding vacuole thickness. For IOLs #1–6, the vacuole with the smallest apparent minor axis in each IOL is identified, as shown in Table 8.

In calculation of vacuole tilt relative to the IOL plane, the thickness is assumed to be negligible. However, through investigation of the distribution of vacuole “tilts,” as well as the minimum measured minor axis, the approximate thickness can be reasonably estimated. In IOLs #1–6, there are 935 total vacuoles identified and measured. From the inferred tilts, assuming negligible thickness, the vacuole oriented closest to perpendicular to the IOL has a calculated tilt of 83.6°. If one assumes that there is an equal probability of a vacuole assuming any orientation, this means that there is a 6.4° range, between 83.6° and 90°, that contains no vacuoles. Assuming that any vacuole tilt or orientation is equally likely, the odds of none of the 935 identified vacuoles being in this range is 1 in 9 × 10^29^. It is more likely that some of the vacuoles are in this range, but their thickness slightly alters the inferred tilt. Assuming equal distributions, there should be approximately 10 total vacuoles oriented within 1 degree of 90° with respect to the IOL. We can therefore assume that the vacuoles with the smallest measured minor axes, shown in Table 8, are in fact these theoretical perpendicular vacuoles, and the measured 5-6 *μ*m minor axes is in fact the thickness.

We can further investigate vacuole thickness in IOL #7. Of the 592 identified vacuoles, the highest calculated “tilt” for a vacuole is 75°. The likelihood of none of the vacuoles having a tilt between 75 and 90 degrees is approximately 1 in 7.5 × 10^46^. As with IOLs #1–6, this discrepancy indicates that vacuoles have a non-negligible thickness affecting the estimated vacuole tilt and that the vacuoles having estimated tilts in the 70-degree range are likely closer to 90°. The measured minor axis for the identified vacuole with 75° estimated tilt is roughly 2 *μ*m; however, several other vacuoles characterized as being in orientation group 4 have measured minor axes closer to 1 *μ*m. This indicates that, for these smaller vacuoles, the thickness is roughly 1 *μ*m, or perhaps a bit smaller. While vacuole thickness likely contributes to scatter by individual vacuoles, the main impact on vacuole characterization as done in this study is likely the mischaracterization of vacuoles that are in group 5 as being in group 4.

Between the uncertainty of vacuole thickness and the implicit error in vacuole measurements, it may be more practical to treat vacuole groups 1 and 2 as a single group and treat vacuole groups 4 and 5 as a single group. As investigated earlier, a vacuole thickness of 5 *μ*m, if not accounted for, essentially sets an upper inferred angle limit of 84° for a 50 *μ*m vacuole. For a smaller vacuole with a 5 *μ*m thickness, this limit is even lower. Combined with measurement inaccuracies, this may very well result in some low-tilt group 5 vacuoles being mischaracterized as group 4, blurring the line between group 4 and group 5. Likewise, a 50 *μ*m group 1 vacuole with 0° tilt may be measured with up to 10% inaccuracy, as shown earlier in Table 5. This could result in a calculated tilt of up to 26°, if one axis is incorrectly measured as 45 *μ*m rather than 50 *μ*m. This potential error blurs the line between group 1 and group 2 vacuoles, making consolidation of these vacuole groups an attractive choice. The decision to consolidate the 5 orientation groups into 3 broader groups would weaken the overall strength of the methodology and characterization, but may be necessary, depending on the accuracy and precision of the equipment and methods used.

Additional consideration must be given to the unusual sizes of vacuoles presented in this study. Most observations of vacuoles note sizes between 2–10 *μ*m, with sizes larger than 20 *μ*m being rare [4–8, 15, 20, 26, 28]. The vacuoles studied here are in some cases larger than 50 *μ*m, with vacuoles smaller than 20 *μ*m being rare. However, there are limited cases and situations in which larger vacuoles, on the order of 100 *μ*m across, have been observed. One noteworthy case observed large vacuoles forming shortly after cataract surgery, requiring immediate explantation. The vacuoles' sizes were not directly measured or reported, but examination of the figures reveals vacuoles are roughly 100 *μ*m across, or perhaps a bit larger [17]. In addition, vacuoles' sizes have been found to be directly related to the osmolarity of the surrounding medium, with low salinity media producing vacuoles up to 200 *μ*m across [9]. The exact procedure used for the formation of vacuoles used in this study is not known, so while the sizes seen here may not be the most commonly reported, they are not unheard of. However, the main purpose of this study is to evaluate individual vacuoles' sizes and orientations as well as catalog characteristics for each of these evaluated vacuoles throughout the lens, and this should be minimally affected by the size of the individual vacuole.

While the test methodology introduced in this study can be demonstrated to work for vacuoles of sizes in the micron and submicron range, considerations must be given to potential limitations of this method for characterizing small-sized vacuoles. For vacuoles of the size studied in IOLs #1–6 or larger, we would not expect any additional issues characterizing vacuoles. However, for smaller vacuoles, certain limitations of this study may manifest. First, because this method requires manual measuring of vacuoles, the magnification on the digital microscope must be high enough for this to be possible. Magnifications of 100x and 150x, which were primarily employed in this study, would likely work for vacuoles down to about 10 *μ*m, albeit with some loss of accuracy. For vacuoles approaching 1 *μ*m across, higher magnifications were demonstrated to be necessary. While 2000x magnification was capable of imaging the individual vacuoles, there is a corresponding loss of FOV. This lower FOV corresponds to only about 0.06% of the total IOL surface or 0.07% of the total IOL volume. Because of this, it would be inaccurate to assume that a few sample images of the IOL would be able to represent the entire lens, especially if the small vacuoles are sparse. In addition, if an IOL has both large and small vacuoles, a magnification large enough to image the small vacuole may be too high to contain a large vacuole in the FOV. While this could limit the ability to generalize characteristics of small vacuoles throughout an IOL, the ability to characterize individual vacuoles should be unaffected regardless of vacuole size.

With a strengthened ability to characterize individual vacuoles, the next step to take is quantitative evaluation of vacuole properties that cause scatter and straylight, particularly that which is expected to affect patients' vision. Extensive work has already been done in this field, most recently by Labuz et al., linking vacuole density and area obscured to C-Quant straylight measurements [18, 28]. However, these studies, like most previous studies, primarily focus on vacuole density and average size [4–7, 13, 14, 17, 20, 26, 32]. Vacuole orientations with respect to the incoming light, however, have not yet been correlated with measured scatter. Further work can be done, using these existing methodologies and results, to link vacuole orientations to scatter.

One additional factor to consider is the direction of scatter by individual vacuoles. As described by van den Berg [22], the point spread function (PSF) defining defocused light can be divided into two domains, these being the central domain caused mainly by aberrations and the peripheral domain caused by straylight and wide-angle scatter. Wide-angle peripheral scatter, as described by van den Berg and by Werner et al. [31], is unlikely to lower VA in any meaningful way, as even a large drop in CS will not affect acuity or visual function. However, close-angle scatter, caused by aberrations or microaberrations, will directly impact VA through distortion of the image projected onto the macula. Further evaluation of scatter caused by vacuoles of differing orientations should be performed to clarify this effect, specifically how orientation affects the angle of scattered light.

That being said, moving forward, it may be beneficial to examine the effects that vacuole orientation groupings have on scatter. One can surmise that orientation of vacuoles of groups 1 and 2 will likely cause the least straylight and scatter, as incoming light normal to the IOL will also be normal to the vacuole, transmitting through with minimal scattering. However, what little light is scattered is also more likely to be in the central domain described by van den Berg [22], resulting in a loss of VA. This phenomenon can be observed in the vacuole images presented in Figure 8; the backlit group 1 and group 2 vacuoles allow light to reach the microscope objective, making them appear white, while the group 4 and group 5 vacuoles divert the light from reaching the objective, making them appear dark. In the same way, the differing scatter potentials of differently oriented vacuoles likely affect light scatter in the eye in different ways, so investigation into the clinical and *in vitro* effects of differently oriented vacuoles should be performed.

## 5. Conclusion

The effects of vacuoles on IOL clarity and light scatter have been extensively studied but mainly in terms of overall density and average size of the individual vacuoles. The orientation of the vacuoles and the distribution of differently oriented vacuoles have not been studied or considered when correlating the scattered light with vacuoles. The present study is focused on a comprehensive quantitative evaluation of IOL samples with varying densities of vacuoles and compared not only the size and densities of the vacuoles present but also the shape and orientation of the vacuoles. A grading scale was developed to compare vacuoles of similar sizes but differing orientations within the IOL. It was shown that similar IOLs with similar vacuole densities can have differing orientations of vacuoles throughout the lens, which should be considered in future studies evaluating scattering by IOL vacuoles.

The presented classification method can be used in future studies of vacuoles, particularly regarding their scattering potential. In addition, digital microscopy could be further refined and utilized to better image IOLs *in vivo*, without requiring explantation. This would allow accurate, noninvasive assessment of vacuole presence, sizes, and orientations, allowing more informed clinical decisions to be made.

## Figures and Tables

**Table 1:** Defined rotation of each vacuole orientation group.

Vacuole orientation group	Rotation angle
Group 1	0–20°
Group 2	20–45°
Group 3	45–60°
Group 4	60–80°
Group 5	80–90°

**Table 2:** The number of vacuoles counted in the stitched images of each IOL and the estimated vacuole count from 100x and 150x images.

IOL #	Total number of vacuoles counted	Estimated number of vacuoles from 100x images	Estimated number of vacuoles from 150x images
#1	470	538 ± 35	599 ± 47
#2	106	127 ± 11	142 ± 32
#3	125	127 ± 10	112 ± 17
#4	68	75 ± 7	81 ± 21
#5	109	111 ± 18	130 ± 29
#6	57	55 ± 9	73 ± 23

**Table 3:** The average vacuole diameter for vacuoles in each IOL and the estimated average vacuole diameter from 150x magnification images.

IOL #	Average vacuole diameter (*μ*m)	Estimated average vacuole diameter from 150x images (*μ*m)
#1	51	51 ± 3
#2	53	54 ± 3
#3	46	55 ± 2
#4	45	52 ± 3
#5	47	54 ± 4
#6	36	38 ± 3

**Table 4:** The total number of vacuoles for each orientation group counted for the three 2000x magnification images of IOL #7, along with the percentage distribution of each group.

Vacuole orientation group	Number counted	Percentage of total (%)
#1	17	3
#2	107	18
#3	227	38
#4	241	41
#5	0	0

**Table 5:** Average dimensions of vacuoles #1–4, along with calculated standard deviation.

Vacuole number	Average measured dimensions (*μ*m)	Standard deviation (*μ*m)	Percent standard deviation (%)
#1	59.2 × 43.8	2.6 × 2.9	4.4 × 6.6
#2	64.8 × 38.5	3.7 × 3.3	5.7 × 8.6
#3	65.1 × 48.6	0.7 × 1.4	1.1 × 3.0
#4	43.0 × 34.0	2.3 × 4.8	5.4 × 14.1

**Table 6:** Average calculated orientations of vacuoles #1–4, along with calculated standard deviation.

Vacuole number	Average orientation angle (degrees)	Standard deviation (degrees)	Percent standard deviation (%)
#1	42.1	3.7	9
#2	53.3	5.4	10
#3	41.7	2.5	6
#4	37.2	9.3	28

**Table 7:** Vacuole orientation group distribution assuming random vacuole orientation.

Vacuole orientation group	Percent distribution (%)	Average measured distribution in IOLs #1–6 (%)
Group 1	22	9 ± 3
Group 2	28	30 ± 4
Group 3	17	25 ± 4
Group 4	22	32 ± 5
Group 5	11	2 ± 2

**Table 8:** The measured major and minor axes for the vacuole with the smallest minor axis length in IOLs #1–6.

IOL #	Major axis length (*μ*m)	Minor axis length (*μ*m)
#1	31	5
#2	45	5
#3	23	6
#4	36	5
#5	36	5
#6	31	6

## Data Availability

The data used to support the findings of this study are available from the corresponding author upon request.

## References

[1] The Eye Diseases Prevalence Research Group (2004). Prevalence of cataract and pseudophakia/aphakia among adults in the United States. *Archives of Ophthalmology*.

[2] Wang W., Yan W., Fotis K. (2016). Cataract surgical rate and socioeconomics: a global study. *Investigative Opthalmology & Visual Science*.

[3] Kawat K., Hayakawa K., Suzuki T. (2012). Simulation of 20-year deterioration of acrylic IOLs using severe accelerated deterioration tests. *Tokai Journal of Experimental and Clinical Medicine*.

[4] Kato K., Nishida M., Yamane H., Nakamae K., Tagami Y., Tetsumoto K. (2001). Glistening formation in an AcrySof lens initiated by spinodal decompositionof the polymer network bytemperature change. *Journal of Cataract & Refractive Surgery*.

[5] Waite A., Faulkner N., Olson R. J. (2007). Glistenings in the single-piece, hydrophobic, acrylic intraocular lenses. *American Journal of Ophthalmology*.

[6] Pérez-Vives C. (2018). Biomaterial influence on intraocular lens performance: an overview. *Journal of Ophthalmology*.

[7] Mönestam E., Behndig A. (2011). Impact on visual function from light scattering and glistenings in intraocular lenses, a long-term study. *Acta Ophthalmologica*.

[8] Gregori N. Z., Spencer T. S., Mamalis N., Olson R. J. (2002). In vitro comparison of glistening formation among hydrophobic acrylic intraocular lenses. *Journal of Cataract & Refractive Surgery*.

[9] Saylor D. M., Coleman Richardson D., Dair B. J., Pollack S. K. (2010). Osmotic cavitation of elastomeric intraocular lenses. *Acta Biomaterialia*.

[10] Hayashi K., Hirata A., Yoshida M., Yoshimura K., Hayashi H. (2012). Long-term effect of surface light scattering and glistenings of intraocular lenses on visual function. *American Journal of Ophthalmology*.

[11] Gamidov A. A., Fedorov A. A., Novikov I. A., Kas’yanov A. A., Siplivyy V. I. (2015). Analyzing causes for opacification of acrylic IOLs. *Vestnik Oftal’Mologii*.

[12] Thomes B. E., Callaghan T. A. (2013). Evaluation of in vitro glistening formation in hydrophobic acrylic intraocular lenses. *Clinical Ophthalmology*.

[13] Xi L., Liu Y., Zhao F., Chen C., Cheng B. (2014). Analysis of glistenings in hydrophobic acrylic intraocular lenses on visual performance. *International Journal of Ophthalmology*.

[14] Schweitzer C., Orignac I., Praud D., Chatoux O., Colin J. (2014). Glistening in glaucomatous eyes: visual performances and risk factors. *Acta Ophthalmologica*.

[15] Rønbeck M., Behndig A., Taube M., Koivula A., Kugelberg M. (2013). Comparison of glistenings in intraocular lenses with three different materials: 12-year follow-up. *Acta Ophthalmologica*.

[16] Yoshida S., Matsushima H., Nagata M., Senoo T., Ota I., Miyake K. (2011). Decreased visual function due to high-level light scattering in a hydrophobic acrylic intraocular lens. *Japanese Journal of Ophthalmology*.

[17] Werner L., Storsberg J., Mauger O. (2008). Unusual pattern of glistening formation on a 3-piece hydrophobic acrylic intraocular lens. *Journal of Cataract & Refractive Surgery*.

[18] Łabuz G., Knebel D., Auffarth G. U. (2018). Glistening formation and light scattering in six hydrophobic-acrylic intraocular lenses. *American Journal of Ophthalmology*.

[19] Hiraoka T., Miyata K., Hayashidera T. (2017). Influence of intraocular lens subsurface nanoglistenings on functional visual acuity. *PLoS One*.

[20] Henriksen B. S., Kinard K., Olson R. J. (2015). Effect of intraocular lens glistening size on visual quality. *Journal of Cataract & Refractive Surgery*.

[21] Matsushima H., Nagata M., Katsuki Y. (2015). Decreased visual acuity resulting from glistening and sub-surface nano-glistening formation in intraocular lenses: a retrospective analysis of 5 cases. *Saudi Journal of Ophthalmology*.

[22] van den Berg T. J. T. P. (2017). The (lack of) relation between straylight and visual acuity. Two domains of the point-spread-function. *Ophthalmic and Physiological Optics*.

[23] Biwer H., Schuber E., Honig M., Spratte B., Baumeister M., Kohnen T. (2015). Objective classification of glistenings in implanted intraocular lenses using Scheimpflug tomography. *Journal of Cataract & Refractive Surgery*.

[24] Kim D.-H., James R. H., Landry R. J., Calogero D., Anderson J., Ilev I. K. (2011). Quantification of glistenings in intraocular lenses using a ballistic-photon removing integrating-sphere method. *Applied Optics*.

[25] Werner L., Morris C., Liu E. (2014). Light transmittance of 1-piece hydrophobic acrylic intraocular lenses with surface light scattering removed from cadaver eyes. *Journal of Cataract & Refractive Surgery*.

[26] van der Mooren M., Franssen L., Piers P. (2013). Effects of glistenings in intraocular lenses. *Biomedical Optics Express*.

[27] Das K. K., Stover J. C., Schwiegerling J., Karakelle M. (2013). Technique for measuring forward light scatter in intraocular lenses. *Journal of Cataract & Refractive Surgery*.

[28] Labuz G., Reus N. J., van den Berg T. J. T. P. (2017). Straylight from glistenings in intraocular lenses: in vitro study. *Journal of Cataract & Refractive Surgery*.

[29] Łabuz G., Vargas-Martín F., van den Berg T. J. T. P., López-Gil N. (2015). Method of in vitro assessment of straylight from intraocular lenses. *Biomedical Optics Express*.

[30] Walker B. N., James R. H., Calogero D., Ilev I. K. (2015). A Novel full-angle scanning light scattering profiler to quantitatively evaluate forward and backward light scattering from intraocular lenses. *Review of Scientific Instruments*.

[31] Werner L., Stover J. C., Schwiegerling J., Das K. K. (2016). Light scattering, straylight, and optical quality in hydrophobic acrylic intraocular lenses with subsurface nanoglistenings. *Journal of Cataract & Refractive Surgery*.

[32] DeHoog E., Doraiswamy A. (2016). Evaluation of loss in optical quality of multifocal intraocular lenses with glistenings. *Journal of Cataract & Refractive Surgery*.

